# Experience in a Climate Simulator: Influence of Probability Function and Feedback on Decisions Against Climate Change

**DOI:** 10.3389/fpsyg.2021.674892

**Published:** 2021-07-14

**Authors:** Gitanshu Choudhary, Varun Dutt

**Affiliations:** ^1^School of Humanities and Social Sciences, Indian Institute of Technology Mandi, Kamand, India; ^2^School of Computing and Electrical Engineering and School of Humanities and Social Sciences, Indian Institute of Technology Mandi, Kamand, India

**Keywords:** wait-and-see preferences, climate microworld, interactive climate change simulator, feedback-availability, investment against climate change, decision aid

## Abstract

Research indicates that people continue to exhibit “wait-and-see” preferences toward climate change, despite constant attempts to raise awareness about its cataclysmic effects. Experiencing climatic catastrophes *via* simulation tools has been found to affect the perception of people regarding climate change and promote pro-environmental behaviors. However, not much is known about how experiential feedback and the probability of climate change in a simulation influence the decisions of people. We developed a web-based tool called Interactive Climate Change Simulator (ICCS) to study the impact of different probabilities of climate change and the availability of feedback on the monetary actions (adaptation or mitigation) taken by individuals. A total of 160 participants from India voluntarily played ICCS across four between-subject conditions (*N* = 40 in each condition). The conditions differed based on the probability of climate change (low or high) and availability of feedback (absent or present). Participants made mitigation and adaptation decisions in ICCS over multiple years and faced monetary consequences of their decisions. There was a significant increase in mitigation actions against climate change when the feedback was present compared to when it was absent. The mitigation and adaptation investments against climate change were not significantly affected by the probability of climate change. The interaction between probability of climate consequences and availability of feedback was significant: In the presence of feedback, the high probability of climate change resulted in higher mitigation and adaptation investments against climate change. Overall, the experience gained in the ICCS tool helped alleviate peoples' “wait-and-see” preferences and increased the monetary investments to counter climate change. Simulation tools like ICCS have the potential to increase people's understanding of climatic disasters and can act as a useful aid for educationalists and policymakers.

## Introduction

Climate change is an urgent issue facing mankind in the present times (IPCC, 2014). The average temperature of the Earth is rising with each passing decade (Lindsey and Dahlman, 2020). This increase in temperature is predicted to increase the occurrences of climatic calamities (CSSR, 2017). There is a consensus that greenhouse gases (GHGs) due to human actions are the leading cause of climate change (Cook et al., 2016). The increases in GHGs are estimated to cause over 150,000 deaths per year (WHO, 2010). Overall, there is an urgent need to take different countermeasures against climate change.

Countering climate change may involve two possible actions: mitigation and adaptation (NASA, 2020). Climate change mitigation refers to the actions taken to reduce the GHG concentrations in the environment to limit the rate of global warming (Hasson et al., 2010). In contrast, climate change adaptation refers to the preventive actions taken to prepare against the catastrophic consequences of climate change (Hasson et al., 2010; UNFCCC, 2020a). Mitigation has been a point of focus ever since the GHG levels started rising at a global level (UNFCCC, 2020b). Despite the fact that the world has already committed to a certain degree of climate change, there have only been limited mitigation efforts (Burton et al., 2007). In the face of the limited success of mitigation measures, adaption measures may provide people with alternate ways to counter the adverse consequences of climate change (UNFCCC, 2020a). For example, climate change adaptation measures may include different types of insurance plans against the different climate change consequences. Overall, as global GHG concentrations increase, there is an urgent need to incorporate both adaption and mitigation measures in the decision-making of people to counter climate change (American Psychological Association, 2009).

The GHG emissions around the world continue to rise (IPCC, 2018), and this increase in emissions could be attributed to people's “wait-and-see” preferences for mitigation and adaptation actions against climate change (Dutt and Gonzalez, 2012a,b; IPCC, 2018). According to these wait-and-see preferences, people are of the opinion that mitigation or adaptation against climate change could be delayed until there are clear signs of a global climate catastrophe (Sterman and Sweeney, 2007; Sterman, 2008; Kumar and Dutt, 2018). Prior research indicates that people's wait-and-see preferences against climate change may be influenced by the future possibility of a climate catastrophe (i.e., the probability of occurrence of a climatic catastrophe in the future) (Dutt and Gonzalez, 2012a) and experiences of such climatic catastrophes (i.e., day-to-day experiences of climatic consequences in the personal life of an individual) (Dutt and Gonzalez, 2012a; Bergquist et al., 2019).

One likely reason for an individual's mitigation and adaptation actions against climate change may be because of one's prior experiences of climatic consequences (Bergquist et al., 2019). For example, research shows that an individual's decisions may differ based on the format in which the information about consequences is collected: experience or description (Dutt and Gonzalez, 2012a; Hertwig, 2012). When the consequences are described *via* a text description in reports, people tend to over-weight a low-probable event and under-weight a high-probable event in their decisions (Dutt and Gonzalez, 2012a; Hertwig, 2012). Conversely, when the consequences are experienced in the world, people tend to over-weight a high-probable event and under-weight a low-probable event in their decisions (Dutt and Gonzalez, 2012a; Hertwig, 2012). Due to the over-weighting of high-probability negative events in the experience, people may increase their pro-environmental actions (Chaturvedi et al., 2017, 2018). For example, in a study unrelated to climate change, Chaturvedi et al. (2017, 2018) used a simulation tool called the Interactive Landslide Simulator (ILS) to study participant's monetary investments toward landslide mitigation. Chaturvedi et al. (2017, 2018) found that participants who were continuously provided with experiences concerning the negative outcomes of their actions due to the landslide calamities in ILS showed an increase in their monetary investments for landslide mitigation compared to those who only read text-based descriptions about the negative landslide outcomes of their actions. Although Chaturvedi et al. (2017, 2018) did attempt to study people's mitigation actions in experience and description formats, their focus was on landslide disasters, and also, these authors did not study the effect of both mitigation and adaptation actions separately.

In a different study, Dutt and Gonzalez (2012a) exposed participants to future climate consequences *via* descriptive and experiential formats, where these consequences were both probabilistic and uncertain in their timing of occurrence. Results revealed that participants exposed to an uncertain occurrence of climate change *via* experience showed more wait-and-see preferences than the group provided with a written description (Dutt and Gonzalez, 2012a). Although these authors experimented with experiential and descriptive formats in a climate problem, these authors did not distinguish between mitigation and adaptation. Furthermore, the experience given was only monetary, and it lacked any kind of imagery.

Literature in climatic catastrophe's experience suggests that personally experiencing a climatic catastrophe can lead to a significant increase in society's risk perception toward climate change (Van der Linden, 2015). A global survey conducted by Gallup world poll revealed that personally experiencing the changing temperatures in the local area was the most crucial factor in raising awareness and concern about climate change among African and Asian countries (McSweeney, 2016). Two independent surveys highlighted that a significant surge in the frequency of climatic-related consequences had shifted the American population's opinions from “is it happening” to a more concerned state for climate change (Revkin, 2019). Thus, a low-probability event generally does not raise as much concern as events with higher chances of occurring. However, in the rare instances that a lower probability event occurs, it attracts much more attention than warranted by its probability (Weber, 2006). Overall, it is interesting to study how the experiences of these lower probability climate events affect people's wait-and-see preferences for mitigation and adaptation actions toward climate change.

Another likely reason for the mitigation and investment actions of an individual against climate change can be the uncertainties in the probabilistic occurrence of a climatic catastrophe in the future (Milinski et al., 2008; Dutt and Gonzalez, 2012a; Kumar and Dutt, 2018). For example, people's wait-and-see preferences for mitigation and adaptation actions may arise from the lack of consensus among the scientific community about the probability and timing of future climatic catastrophes (Nordhaus, 1994). Nordhaus (1994) performed a series of interviews with social and natural scientists. It was found that there was a large variation in the probability estimates among the scientific community (Nordhaus, 1994).

Furthermore, the pace of climate change, which is accelerating from its prior estimates, causes uncertainties in climate change consequences (Fountain, 2019). For example, the Canadian Arctic permafrost had already melted in the year 2016 to levels that were not expected until 2090 (Farquharson et al., 2019). Beyond the uncertainties in the probability distributions of future climate catastrophes, people also seriously underestimate the actual non-linear increase in the accumulation, which further contributes to strengthening their wait-and-see preferences (Dutt and Gonzalez, 2012b). Overall, it is interesting to evaluate how the uncertainties in the probabilities of climate change catastrophes influence the mitigation and adaptation actions of people.

Motivated by the above observations, this research aims at understanding the effects of availability of experience (feedback) or its absence (description) and the different probabilities of climate change occurrence on the mitigation and adaptation decisions of people toward climate change. More precisely, we examine whether participants increase their monetary investments in mitigation or adaptation in experience formats compared to description formats. Additionally, we examine how the different probabilities of climate change influence the monetary mitigation or adaptation actions of people. A web-based simulation tool called the Interactive Climate Change Simulator (ICCS) is developed in this research to study the monetary investment patterns of individuals in climate change mitigation and adaptation. The ICCS tool allows participants to invest money in climate change mitigation and adaptation, and it provides imagery feedback to participants about the probabilistic climate change consequences. In the ICCS tool, mitigation investments reduce the probability of climate change, whereas adaptation investments reduce the consequences of climate change. Overall, the ICCS overcomes some of the stated limitations on the saliency of feedback in prior literature cited above. To the best of our knowledge, the proposed ICCS tool is the first of its kind, which allows researchers to investigate the impact of feedback and probability of climate change on the mitigation and adaptation actions of people. The tool could be used for both climate education and climate policymaking.

In what follows, we first summarize the existing literature relevant to generating our hypothesis about the probability of climate change occurrence and the availability of feedback. Next, we present the design and working of the ICCS tool, which was developed to study the individual's mitigation and adaptation decisions against climate change. Finally, we present the findings of our experiment and discuss the implications of our results to educators and policymakers.

## Background

Prior literature concerning climate change has made it clear that personally experiencing a climatic consequence leads to greater concern toward climate change and a greater willingness to invest against future climatic catastrophes (Weber, 2006; Spencer et al., 2011; Lang and Ryder, 2016; Demski et al., 2017; Kumar and Dutt, 2018, 2019; Bergquist et al., 2019). For example, Spencer et al. (2011) conducted a national survey following a flood calamity in the United Kingdom. Results showed that having experienced a flooding calamity firsthand resulted in a more significant concern toward climate change, which resulted in a greater willingness to take mitigation action against climate change. Similarly, Bergquist et al. (2019) conducted a survey on a selective sample of participants who had recently experienced Hurricane Irma. The study revealed that experience mattered as experiencing Hurricane Irma intensified the sample's negative emotions toward climate change.

Furthermore, studies conducted in laboratory settings have found that induced feedback in simulation tools may also increase participant's concern toward climate change and help participants improve their understanding of the underlying system dynamics (Dutt and Gonzalez, 2012b; Chaturvedi et al., 2017, 2018). For example, Dutt and Gonzalez (2012b) developed a dynamic climate change simulator (DCCS) tool to study the effects of experimental feedback on the misconceptions of people concerning the Earth's climate. Results revealed that feedback in DCCS helped alleviate people's cognitive misconceptions concerning the functioning of the climatic system compared to a no-DCCS intervention (Dutt and Gonzalez, 2012b; Kumar and Dutt, 2018). Based upon the above literature, we hypothesize that:

*H1*: Investments in mitigation and adaptation will be greater in conditions when feedback is present compared to when feedback is absent.

Furthermore, the probability of climate change and its consequences have great variability (IPCC, 2018). For example, there exist a number of emission scenarios related to Earth's climate, where some of these scenarios range from being optimistic to being pessimistic (IPCC, 2000). The variability in climate change consequences is also exhibited by both climate and social scientists (Nordhaus, 1994). For example, in interviews conducted by Nordhaus (1994), the opinions of the social and natural experts differentiated based upon the economic consequences of climate change as well as the future probability of climate change.

Prior research in decision-making shows that the variability in climate probability or its consequences makes people respond differently to climate change (Weber, 2006; Milinski et al., 2008; Hasson et al., 2010). As per Weber (2006), the higher the probability of a calamity, the more concern, and attention people pay to it. Similarly, Milinski et al. (2008) showed that in a collective-risk-social-dilemma game, a high 90% probability of climate change caused several groups to reach the target compared to a low 10% probability of climate change. Hasson et al. (2010) also provided insights into the human tendency to pay more attention and consequently respond to climatic situations with a higher possibility of loss.

Furthermore, prior research in behavioral economics reveals that people over-weight small probability events when these events are presented descriptively (without feedback) and people under-weight small probability events when these events are experienced *via* feedback (Kahneman and Tversky, 1979; Hertwig and Erev, 2009; Hertwig, 2012). Inversely, a high-probability event is over-weighed *via* feedback and under-weighted when described in a written form (Dutt and Gonzalez, 2012a; Hertwig, 2012). Based upon this literature, people would tend to increase their investments in conditions where they experience high-probability climate consequences *via* feedback compared to conditions where they experience low-probability climate consequences. Furthermore, people would tend to decrease their investments in conditions where they read written descriptions of high-probability climate consequences compared to conditions where they read written descriptions of low-probability climate consequences. Overall based upon the above literature, we hypothesize that:

*H2:* There would be an interaction between the probability of climate consequences (high or low) and the feedback (present or absent).

Next, we detail a simulation environment, the ICCS, which was used to test the above-stated hypotheses in an experiment involving human participants.

## The ICCS Model

The ICCS model is adapted from the model suggested by Chaturvedi et al. (2017, 2018). In the ICCS model, we simulate the cataclysmic effects of climate change based on human monetary investments in mitigation and adaptation. Thus, participants can reduce the probability of a climatic disaster by investing in mitigation actions. In contrast, not investing in mitigation actions may increase the probability of climatic change disasters. Additionally, we allow participants to adapt to the cataclysmic effects of climate change. Investment in adaptation does not influence the probability of climate change occurrence; instead, the cost of the disaster is influenced. The ICCS model enables participants to invest in adaptation in the form of three different insurance schemes, namely, life insurance against fatality, health insurance against injury, and property insurance against property damage. Investment in these insurance schemes reduces the corresponding monetary loss incurred in case a climatic disaster occurs. In ICCS, climatic disasters occur in the form of cyclones, floods, and droughts. These three calamities were selected based on the Indian topography, where the present study was carried out (Nair et al., 2013). The occurrence of climate change and its corresponding disaster(s) was simulated for 36 trials in the ICCS, where each trial corresponded to a year in the ICCS model. Thus, climate change was simulated over a period of 36 years in the ICCS model.

The ICCS model first computes the probability of a climate change disaster based upon the amount invested in mitigation. Thus, the decision of a participant to mitigate climatic change is used to compute the probability of a climatic disaster for a particular trial.

### Probability of Climate Change

As discussed above, the probability of climate change disaster in the ICCS model is a function of the participant's investment in mitigation. The following function determined the probability of a climate change disaster taking place:

(1) $$p=1-m*{\left(\frac{\sum_{t=1}^{n}{\text{investment}}_{\text{t}}}{\sum_{t=1}^{n}{\text{income}}_{\text{t}}}\right)}^{k}$$

where *p* is the probability of a climate change disaster; *m* is the return on the mitigation amount; the $\overset{n}{\underset{t=1}{\sum }}investment_{t}$ is the sum of investments made in mitigating climate change between the first year and the current (*n*^*th*^) year; the $\overset{n}{\underset{t=1}{\sum }}income_{t}$ is the sum of income available for making investments between the first year and the current (*n*^*th*^) year; and *k* is the exponent parameter.

According to Equation (1), the probability of climate change depended on the mitigation investment decisions of an individual. The more monetary assets a participant dedicated to mitigation, the lower was the probability of climate change disasters. In the ICCS model, a climate change disaster was simulated when the probability of climate change disaster *(p)* was greater than or equal to a uniformly distributed random number [~ *U* (0,1)] for a particular year.

### Damage Due to Climate Change Disasters

The damages due to climate change disasters in the ICCS model were classified into three independent categories: injury, fatality, and property loss. Based upon the nature of climate problem, cyclones and floods simulated damages due to injury, fatality, and property loss; however, droughts simulated damages in the form of injury and fatality. Each damage had an independent probability of causing loss within the ICCS model, where it was not necessary that all disasters would lead to losses. As in the real world, when disasters occur, they could be catastrophic or harmless. A catastrophic disaster is one that resulted in injury or damages to life and property. A climatic disaster became catastrophic in the ICCS model based on the damage probability value assigned to injury, fatality, and property loss. Thus, when a uniformly distributed random number was less than or equal to the corresponding damage probability, the corresponding damage was assumed to occur within the ICCS tool. The occurrence of all three damage modes, injury, fatality, and property damage, was possible simultaneously. In contrast, if the generated random numbers were more than the corresponding probability of damages, then the corresponding climate change disasters turned out to be harmless. The exact probability values of injury, fatality, and property damages are detailed ahead in this paper.

### Adaptation Against Climate Change Disasters

In the ICCS model, the availability of adaptation schemes helps deal with the catastrophic consequences of climate change by reducing the amount of loss incurred due to the disasters. As mentioned above, there existed three insurance schemes to prepare against the potential damages possible due to climate change, which worked as adaption measures in the ICCS model. Health insurance helped minimize the loss incurred due to injury damage. Similarly, life insurance and property insurance helped reduce the loss due to fatality and property damage, respectively. Thus, in the ICCS model, the function of insurance schemes was to reduce the amount of loss incurred due to injury, fatality, and property damages.

If a damage condition was simulated due to climate change and the participant had purchased the corresponding insurance scheme, then it led to the computation of a reduced percentage of loss in the ICCS model. The attenuated percentage of loss was calculated based on the number of times the participant had enrolled in a particular insurance scheme. The following equation was used for the computation of loss percentage:

(2) $$r=\left(\frac{100}{T}\right)*\left(\sum_{t=1}^{n}{\text{insurance}}_{t}\right)$$

where *r* is the percentage of loss, when insurance was bought; *T* is the total number of trials in the ICCS; $\overset{n}{\underset{t=1}{\sum }}insurance_{t}$ is the sum of the number of times insurance was bought between the first year and the current (*n*^*th*^) year. For example, if property damage were to occur and the participant had purchased property insurance once in each year for the first 5 years in a game lasting 36 years, then the percentage of loss due to property damage would be (5/36) ^*^ (100), or 13.88%.

The loss incurred when a participant had invested in an insurance scheme was capped such that the loss percentage would be at most when no such insurance investment was made. Therefore, an upper limit was set on the percentage of losses due to insurance investment for injury, fatality, and property damages.

## The Iccs Tool

The ICCS tool is a web-based tool developed to study individuals' monetary investment patterns against climate change, which is based on the ICCS model. The ICCS tool is programmed in open-source programming languages, PHP (version 7.2) and MySQL (version 5.7). The tool enables participants to make repeated monetary investment decisions against climate change by mitigating against or adapting to climate change, and it provides participants feedback regarding the outcome of their investment decisions. The ICCS tool allows people to try various monetary approaches toward climate change and experience the associated consequences in order to help people better comprehend the impact of climate change. Different parameters within the ICCS tool are completely customizable. For example, the ICCS tool could run for a total of N trials, where each trial can be expressed in a certain unit of time (e.g., one trial could be a year). The participants are given a starting annual income and an initial property wealth in an imaginary currency. At any given point in time, a participant's total wealth is the sum of his remaining property wealth and the income not invested against climate change. The goal of the ICCS tool is to maximize one's total wealth.

In the ICCS tool, the total wealth of participant is dependent on the investments of an individual in mitigation and adaptation and the negative climatic outcomes. The total wealth could decrease as a result of injury, fatality, and property damages. The ICCS tool allows participants to invest in mitigation, adaptation, or both within a trial to deal with the changing climatic conditions and resulting disasters. As described above, adaption against climate change is carried out in the form of insurance schemes. The three insurance schemes available to adapt to climate change in the ICCS tool are chargeable to the income of the participant, and once brought, they are only applicable for the subsequent trial. In a trial, participants could invest in any or all insurance schemes as long as they had the necessary income to do so. Beyond insurance, participants were free to choose how much they wanted to invest in mitigating climate change. The maximum possible investment in any trial was the participant's remaining annual income at that instance in the ICCS tool.

**Figure 1:**
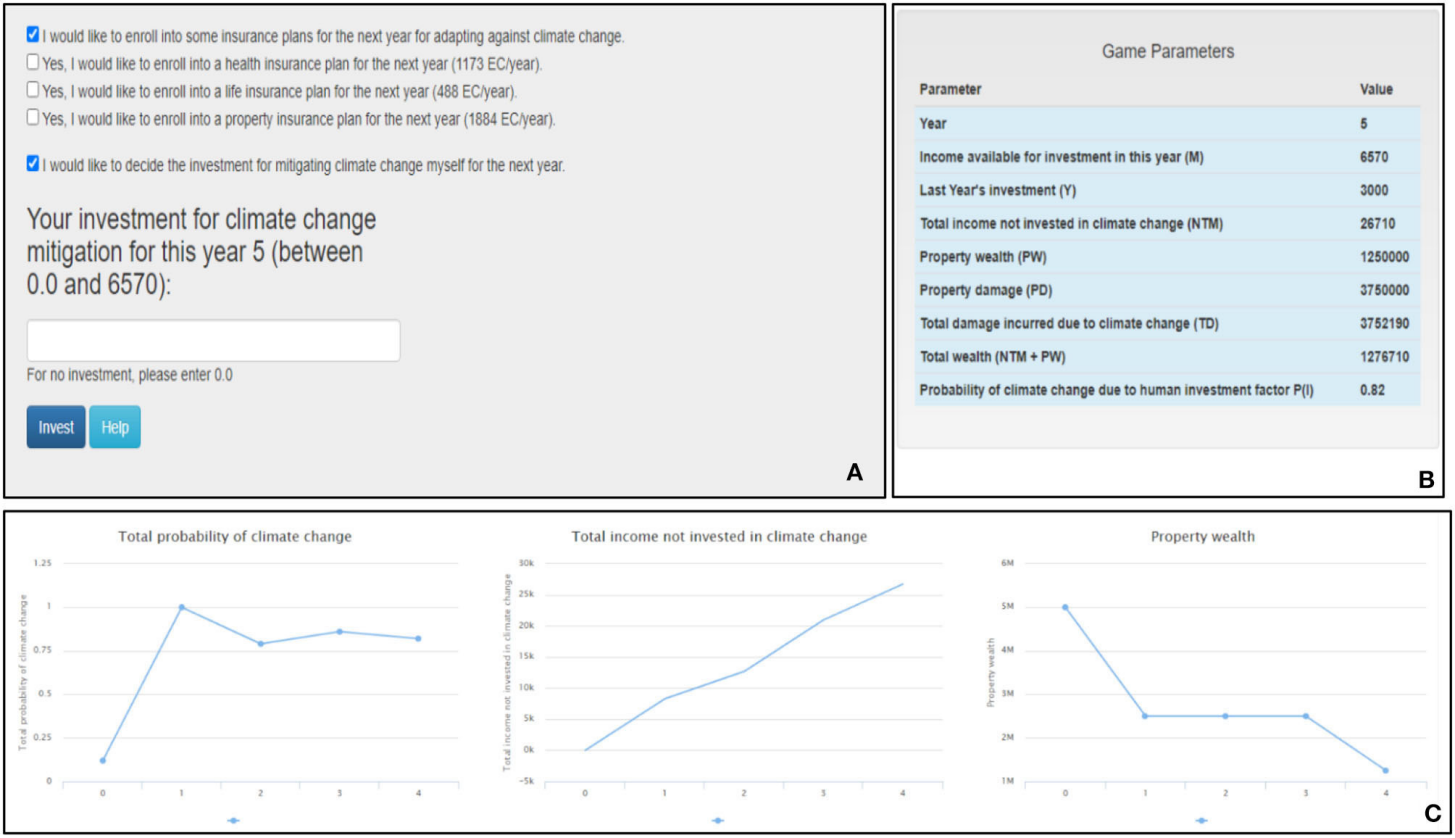
The ICCS tool's investment screen. **(A)** Participants make investments against climate change. **(B)** The tool's different parameters and their values. **(C)** Line graphs showing the total probability of climate change, the total income not invested in climate change and the property wealth over years.

The first screen in the ICCS tool is the monetary investment screen (refer to Figure 1). Panel Figure 1A of the screen is where the participants' monetary mitigation and adaptation decisions are collected. Participants can choose whether they want to mitigate climate change or enroll in an insurance scheme to reduce the possible losses (or both). Figure 1B displays the various parameters of the game and their associated values at that particular instance in the game, including the anthropogenic probability of climate change taking place in the subsequent turn, the remaining annual income, the property wealth, and the total income. This panel helps participants know about the likelihood of disasters taking place and the income available to them at that time. Furthermore, Figure 1C graphically depicts the probability of climate change taking place and its variation from the game's beginning. It also depicts the amount not invested toward climate change mitigation and adaptation, which results in an increase in the total wealth of the participants. The stimuli provided on the screen help participants decide their future course of action, and once they have locked in their monetary investments, they click the “Invest” button, which leads them to the second screen.

**Figure 2:**
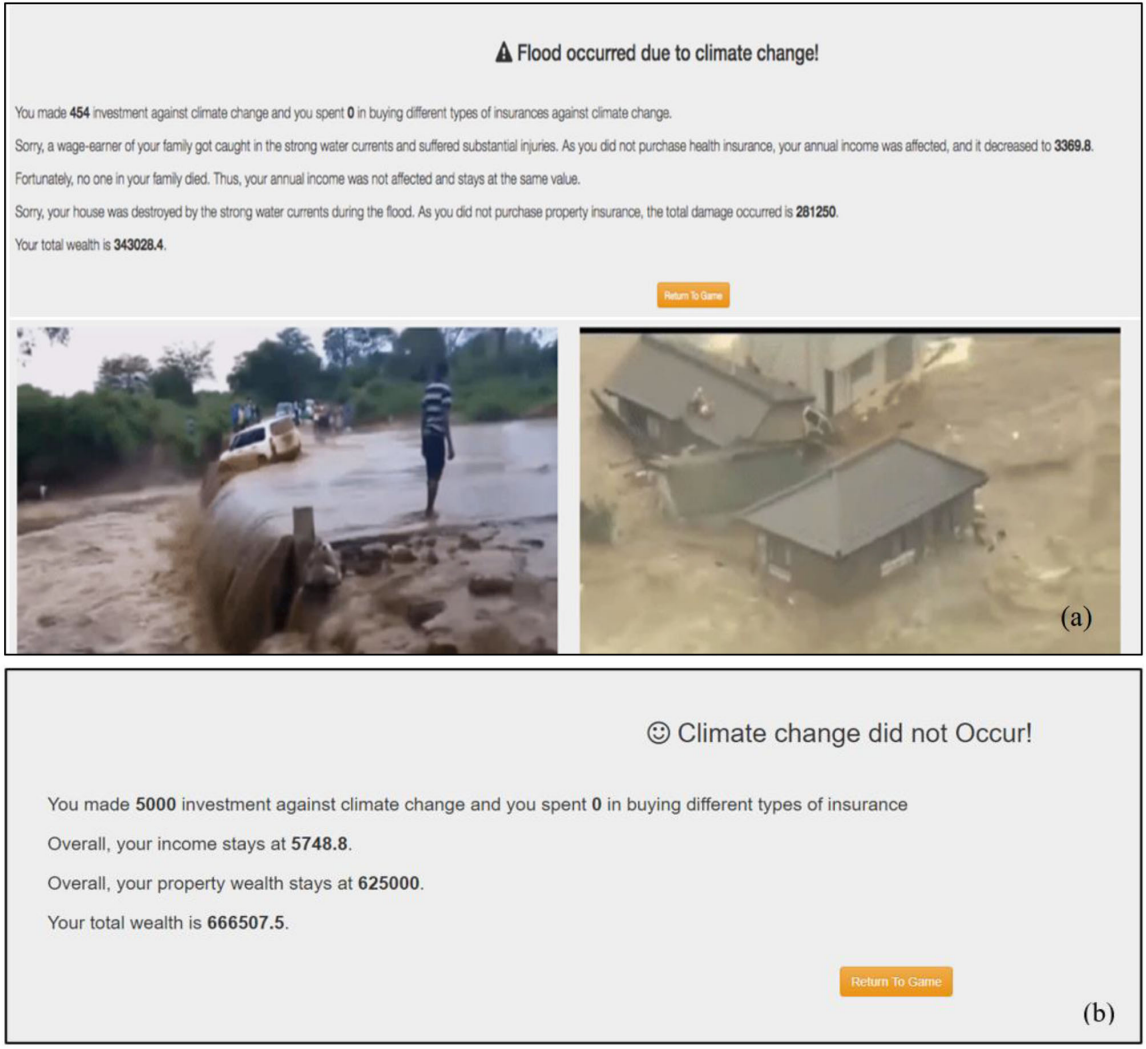
Feedback screens. **(a)** Negative feedback screen, which informs participants about the losses incurred due to climatic disasters. **(b)** Positive feedback screen, which informs participants that climatic disasters did not occur.

The second screen is the feedback screen (refer to Figure 2). Depending on the investment in mitigation, which could vary between zero (minimum) and player's current annual income (maximum), the occurrence of climate change is determined, and the resulting feedback is provided to participants. Figure 2a shows a negative feedback screen. It is generated in the event of a climate change disaster. The screen first depicts what climate change disaster took place (e.g., a flood occurred). Second, the screen provides information associated with the monetary investments and the loss of wealth due to the damages incurred. Finally, the screen shows the images corresponding to the incurred damages. Figure 2b represents a positive feedback screen, and it is generated when no disaster occurs. The screen allows participants to know that climate change did not occur for the current trial. Also, the screen informs participants about their remaining wealth and their investment in the current trial toward climate change. Clicking the “Return to Game” button leads participants back to the investment screen for the next round.

## Experiment: Influence of Feedback and Probability of Climate Change

We performed an experiment to test the effectiveness of feedback availability and the probability of a climate change disaster. The data from the study are available upon request to the authors.

## Methodology

### Experimental Design

A total of 160 participants were randomly assigned across four between-subject conditions in the ICCS tool, where each condition had 40 participants. The conditions differed based on the availability of the feedback (feedback present or feedback absent) and the probability of climate change [high (cubic) probability or low (linear) probability]. The linear and cubic probability functions involved *k* = 1 and *k* = 3 in Equation 1, respectively. The linear probability would cause a slow (linear) change in probability; however, the cubic probability would cause a rapid (non-linear) change in probability. Based upon the probability of climate change and feedback, the four between-subject conditions were linear feedback (*N* = 40), linear no-feedback (*N* = 40), cubic feedback (*N* = 40), and cubic no-feedback (*N* = 40).

As mentioned in the ICCS model section, each condition in the ICCS was 36 trials long (where one trial was equal to 1 year). In each trial, participants were asked to invest against climate change using the income at their disposal. Participants were given a starting annual income of 8,760 EC and the property wealth of 50,000,00 EC (EC being an imaginary currency). In each condition, participants' goal was to maximize their total wealth through investments over the course of the ICCS performance. As mentioned above, within the ICCS tool, feedback regarding climate change occurrence was provided in the form of cyclones, floods, and droughts. Due to climate change, cyclones, droughts, and floods could occur with 33, 33, and 34% chance, respectively, in a trial. If any of the climate change consequences were to occur, then the probability of incurring losses due to injury, fatality, and property damages was set at 30, 9, and 50%, respectively. Climate change consequences could occur independently of each other.

In conditions with feedback, participants were provided with numerical and imagery feedback regarding the outcomes of their investment activities (refer to Figures 2a,b). They were also provided with graphical information about the probability of climate change, monetary assets, and property wealth on the investment screen to better comprehend the effects of their investment behavior against climate change (refer to Figure 1). In contrast, participants in feedback-absent conditions were not provided with any graphical information on the investment screen or feedback concerning their actions. Instead, they were given a written text description about the climate change problem, and they were asked to make investments based on it. More precisely, even though the participants in feedback-absent conditions were provided with a text explaining how the probability of climate change changes within the ICCS tool (the text description is available in the Supplementary Material), they were not shown the feedback screen or the graphs on the investment screen.

The other independent variable was the probability of climate change. Participants subjected to the cubic probability function (Equation 1 with *k* = 3) faced a higher probability of climate change within the ICCS than participants subjected to the linear probability function (Equation 1 with *k* = 1). We simulated the cubic and linear functions one thousand times to investigate the resulting nature of the climate change probabilities. For these simulations, a binary random function with a 50% chance of buying insurance and a 50% chance of not buying insurance was developed and used. The amount invested in mitigation was determined using a uniformly distributed function ranging between zero (no mitigation) and the available annual income after paying for adaptation. The monetary parameters were the same as in the ICCS tool, with the starting annual income of 8,760 EC and the property wealth of 50,000,00 EC (EC being an imaginary currency). The return to mitigation (*m*) was taken to be 0.85, and the obtained probabilities in Equation 1 were compared against a uniformly distributed random number [~ U (0,1)] to simulate climate change. If the probability of climate change was more than the uniformly distributed random number, then climate change occurred; else, it did not occur.

**Figure 3:**
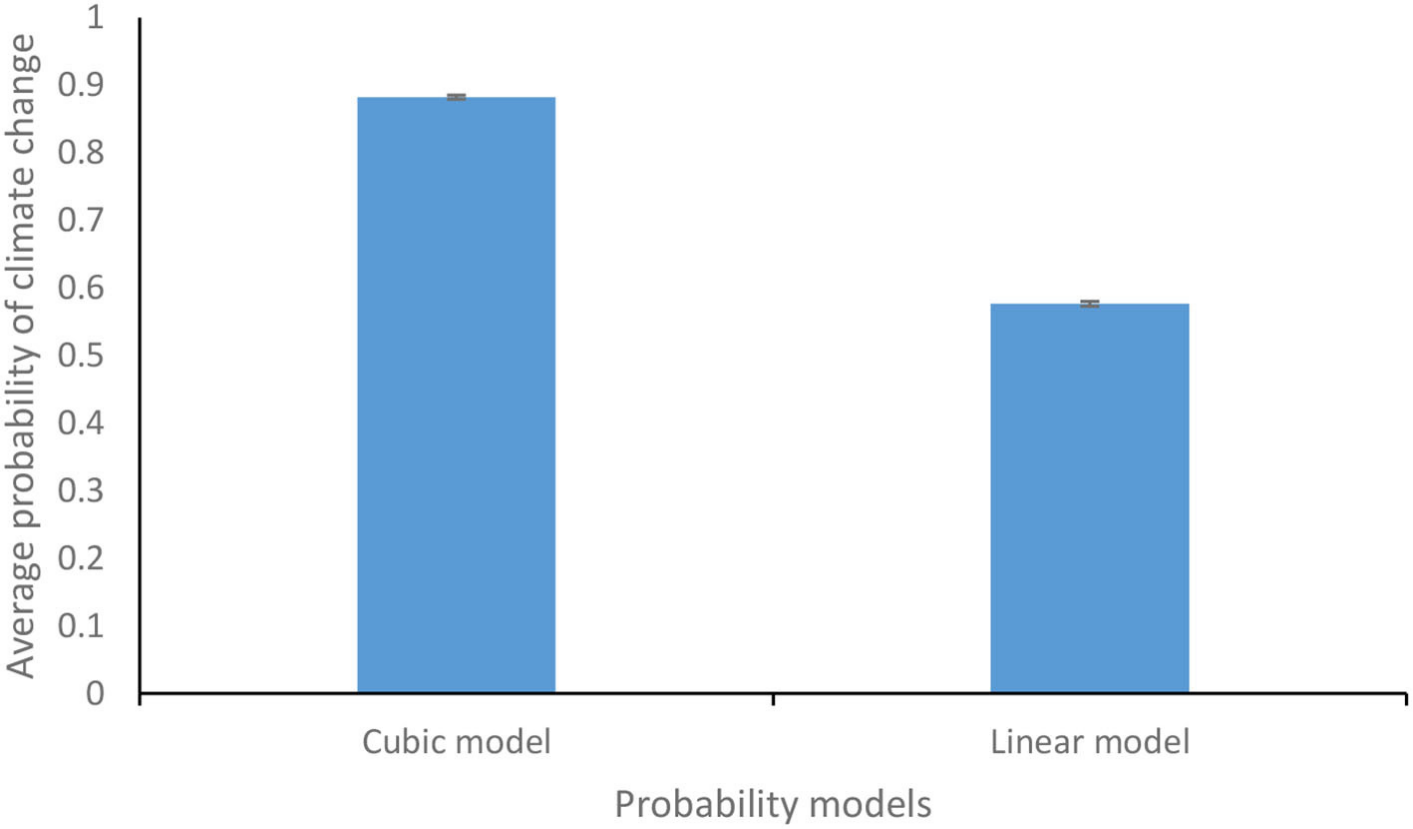
Average probability of climate change for the cubic and the linear functions. The error bars show 95% CI around the point estimate.

Similarly, the occurrence of losses due to climate change was determined by comparing the probability values of injury, fatality, and property damage losses with the corresponding uniformly distributed random number [~ U (0,1)]. If losses occurred, then the available monetary assets were adjusted based on the type of damage incurred. If insurance was bought, then the loss percentage was determined based upon Equation 2. Figure 3 shows the difference between the average probability of climate change for the cubic and linear model. The values obtained by simulating the cubic and linear models one thousand times were averaged to obtain the average probabilities of climate change for the cubic and linear model. As can be inferred from Figure 3, the cubic model resulted in a higher average probability of climate change than the linear model. The participants in the feedback-present conditions were not aware of the exact form of Equation 1, as they experienced the resulting climate consequences. However, participants in the feedback-absent condition were made aware of the exact form of Equation 1 in a descriptive (textual) form, and they did not experience the climate consequences of their monetary actions.

The property wealth was reduced by 50% every time property damage occurred. Similarly, there was a decrement of 12.5 and 25% in the latest annual income of the participant due to injury and fatality damages. These losses were incurred in case the participant had not enrolled in the corresponding annual insurance schemes. The insurance schemes worked to attenuate the losses incurred due to climate change, where the percentage loss due to insurance was determined per Equation 2. Thus, the percentage of loss incurred gradually increased with an increase in the number of times insurance was bought by participants. Overall, the upper limits for percentage loss under different insurance schemes were set as 10, 20, and 50% for injury (health insurance), fatality (life insurance), and property damages (property insurance), respectively.

As already mentioned, the ICCS tool gave participants four options to invest their monetary resources: the three independent insurance (adaptation) schemes (property insurance, health insurance, and life insurance) and the option to invest in mitigation (see Figure 1A). To obtain the performance metrics, participants' monetary investments across the four options, i.e., the three insurance schemes and the option to invest in mitigation, were converted into respective ratios. Thus, we obtained three ratios for adaption schemes: property insurance ratio, health insurance ratio, and life insurance ratio.

A 1.0 represented that the respective insurance was brought for a particular trial. In contrast, a 0.0 signified that the participant did not enroll in a particular insurance scheme. The fourth investment ratio was the mitigation ratio, and it was obtained by computing the money invested in mitigation divided by the total money available to invest at that instance. The fifth ratio was for the total investments in insurance across all three insurance schemes in a particular trial, i.e., the total insurance ratio. It was obtained by dividing the total money invested in insurance schemes in a particular trial by the total amount available to invest at that instance. Similarly, a sixth ratio was obtained for the overall investment across all three adaptation options and mitigation in a particular trial (i.e., total investment ratio). The total investment ratio was computed by dividing the total monetary investment made in a particular trial by the amount of money available to invest at that instance.

### Participants

The study was conducted after approval from the Ethics Committee at the Indian Institute of Technology Mandi, India, with signed written consent from all participants. Participants were randomly recruited from across India through a crowdsourcing site, Amazon Mechanical Turk (Mason and Suri, 2012). Seventy-two percent of the participants were males, and the rest were females. Participants' age ranged from 18 to 52 years (mean = 30.09 years, *SD* = 5.72 years). Among the participants, 68% had completed an undergraduate degree, while the rest had completed a master's degree. Also, 71% of the participants had a science, technology, engineering, and mathematical (STEM) background, and 29% of participants were from a non-STEM background. All the participants were paid INR 30 (USD 0.4) as a participation fee. The average time to complete the study by all the participants was 27 min.

### Procedure

Participants were given a specific set of instructions before the beginning of their experiment (please see the Supplementary Material for the text of instructions given to participants in the feedback-present and feedback-absent conditions for the linear probability function). Participants were also made to play an unrelated scenario in the ICCS for two trials, where they were instructed to make investment decisions in the ICCS tool. Participants had a choice to invest in mitigation, adaptation, or both in each trial. Participants were requested to complete all trials presented to them. Upon successful completion of the 36th trial, participants were thanked and paid for their contribution and time.

### Data Analyses

We used ANOVA to test differences between two or more means (Field, 2013). The conditions of normality, sphericity, and homogeneity are needed to be met for performing ANOVA. The Q–Q plots (between expected quantiles and normal quantiles) showed that the six dependent variables were normally distributed. Furthermore, Mauchly's test of sphericity was found to be significant for the six dependent variables, and therefore, the conditions of sphericity were not met for any of the six dependent variables: total investment ratio [$\chi_{\left(629\right)}^{2}$ = 2377.60, *p* <0.01], mitigation ratio [$\chi_{\left(629\right)}^{2}$ = 2706.74, *p* <0.01], total insurance ratio [$\chi_{\left(629\right)}^{2}$ = 2516.58, *p* <0.01], property insurance ratio [$\chi_{\left(629\right)}^{2}$ = 1613.55, *p* <0.01], health insurance ratio [$\chi_{\left(629\right)}^{2}$ = 1309.96, *p* <0.01], and life insurance ratio [$\chi_{\left(629\right)}^{2}$ = 1206.09, *p* <0.01]. Therefore, Greenhouse–Geisser-corrected degrees of freedom were used to assess the significance of dependent variables. Levene's test showed that variances were homogeneous for the dependent variables over a majority of trials. Therefore, ANOVA was used to test the differences between two or more means (Field, 2013). The alpha level was set at 0.05 (or 5%). We performed a 2 feedback conditions ×2 probability conditions ×36 trial mixed-factorial ANOVA to study the effect of feedback and the probability of climate change on the six investment ratios.

## Results

### Influence of Feedback and Probability

**Figure 4:**
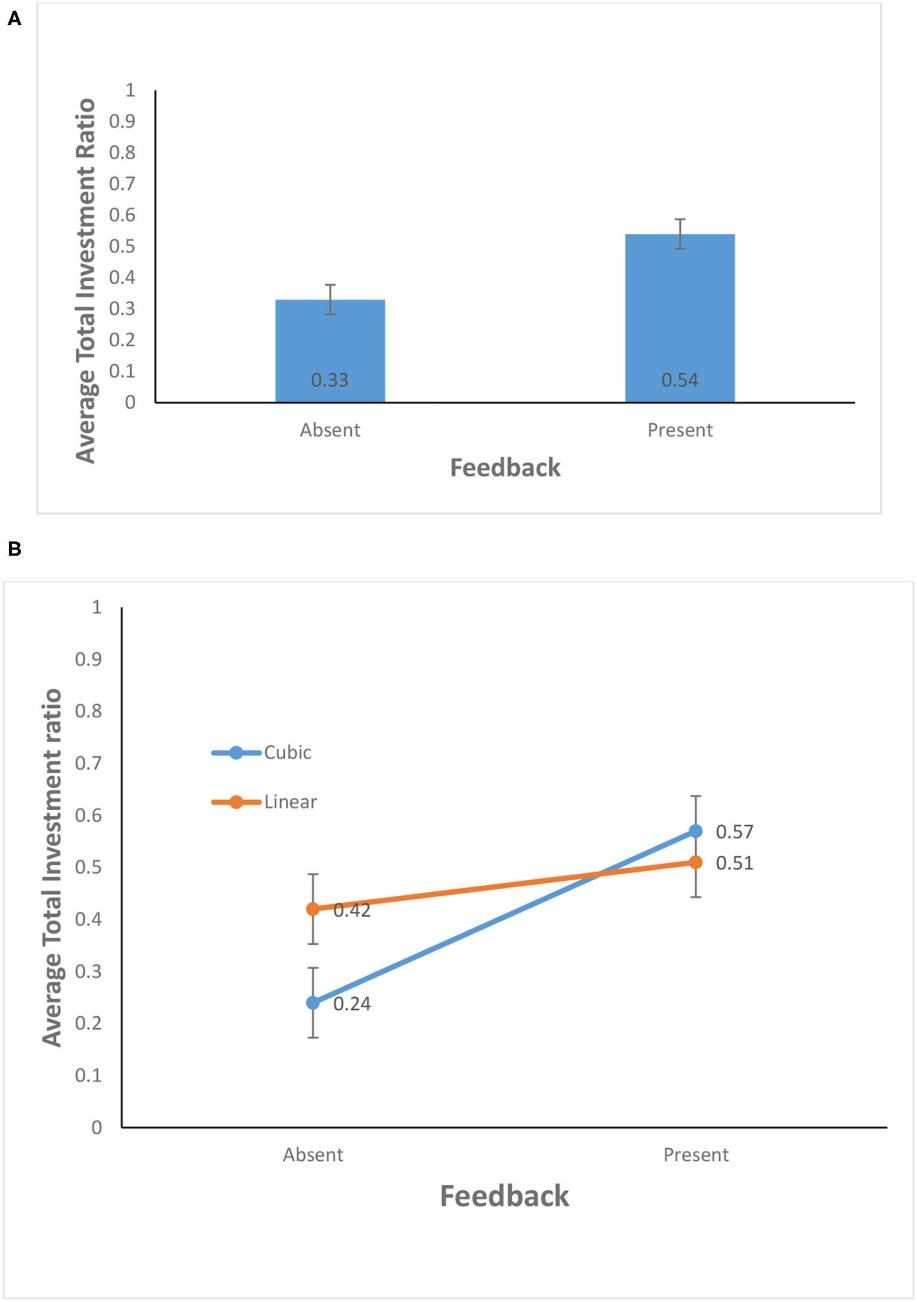
**(A)** Average total investment ratio in feedback-present and absent conditions. **(B)** Average total investment ratio in cubic and linear probability of climate change and feedback-present or absent conditions. The error bars show 95% CI around the point estimate.

As shown in Figure 4A, the average total investment ratio for the feedback-present condition (0.54) was higher than for the feedback-absent condition (0.33) [*F*_(1, 156)_ = 38.43, *p* <0.01, η^2^ = 0.20]. This finding supports H1. In contrast, the probability of climate change did not yield a significant effect: The average total investment ratio for the cubic probability (0.40) was about the same as that for the linear probability (0.47) [*F*_(1, 156)_ = 3.35, *p* = 0.07, η^2^ = 0.02]. Furthermore, as shown in Figure 4B, the interaction between the availability of feedback and probability of climate change was significant for average total investment ratio [*F*_(1, 156)_ = 12.27, *p* <0.01, η^2^ = 0.07]. This finding supports the H2.

**Figure 5:**
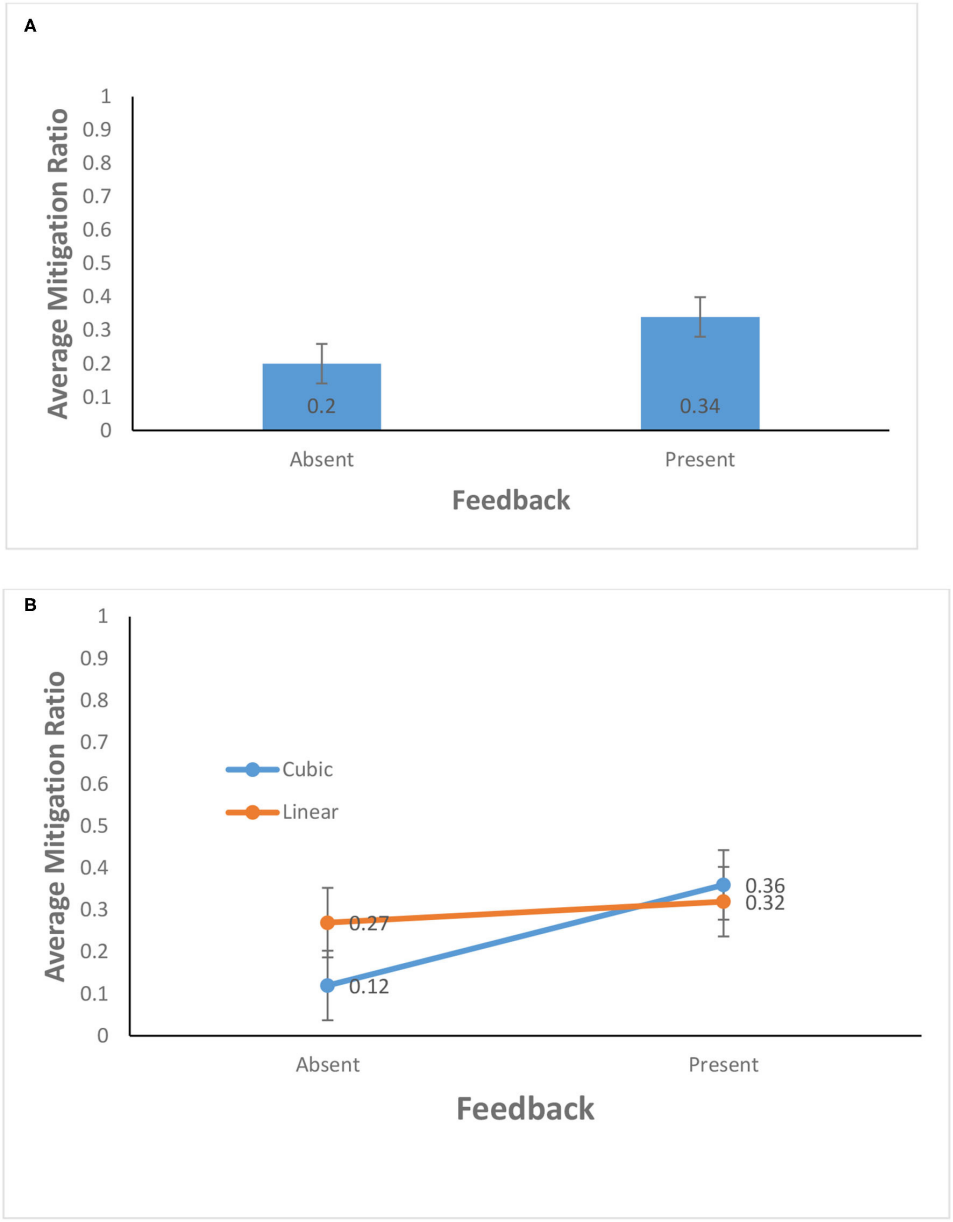
**(A)** Average mitigation ratio in feedback-present and absent conditions. **(B)** Average mitigation ratio in cubic and linear probability of climate change and feedback-present or absent conditions. The error bars show 95% CI around the point estimate.

As shown in Figure 5A, the average mitigation ratio in the feedback-present condition (0.34) was higher than that in the feedback-absent condition (0.2) [*F*_(1, 156)_ = 11.80, *p* = 0.01, η^2^ = 0.07]. This finding supports H1. In contrast, the probability of climate change did not yield a significant effect: The average mitigation ratio for the cubic probability (0.24) was about the same as that for the linear probability (0.30) [*F*_(1, 156)_ = 1.81, *p* = 0.18, η^2^ = 0.01]. Furthermore, as shown in Figure 5B, the interaction between the availability of feedback and probability of climate change was significant for the average mitigation ratio [*F*_(1, 156)_ = 5.13, *p* = 0.03, η^2^ = 0.03]. This finding supports H2.

The average total insurance ratio for the feedback-present condition (0.39) was about the same as that for the feedback-absent condition (0.38) [*F*_(1, 156)_ = 0.06, *p* = 0.80, η^2^ = 0.00]. This finding does not support H1. Similarly, the average total insurance ratio for the cubic probability (0.36) was about the same as that for the linear probability (0.40) [*F*_(1, 156)_ = 0.78, *p* = 0.38, η^2^ = 0.01]. The interaction between the availability of feedback and probability of climate change was not significant for average total insurance ratio [*F*_(1, 156)_ = 2.68, *p* = 0.10, η^2^ = 0.02]. This finding does not support H2.

**Figure 6:**
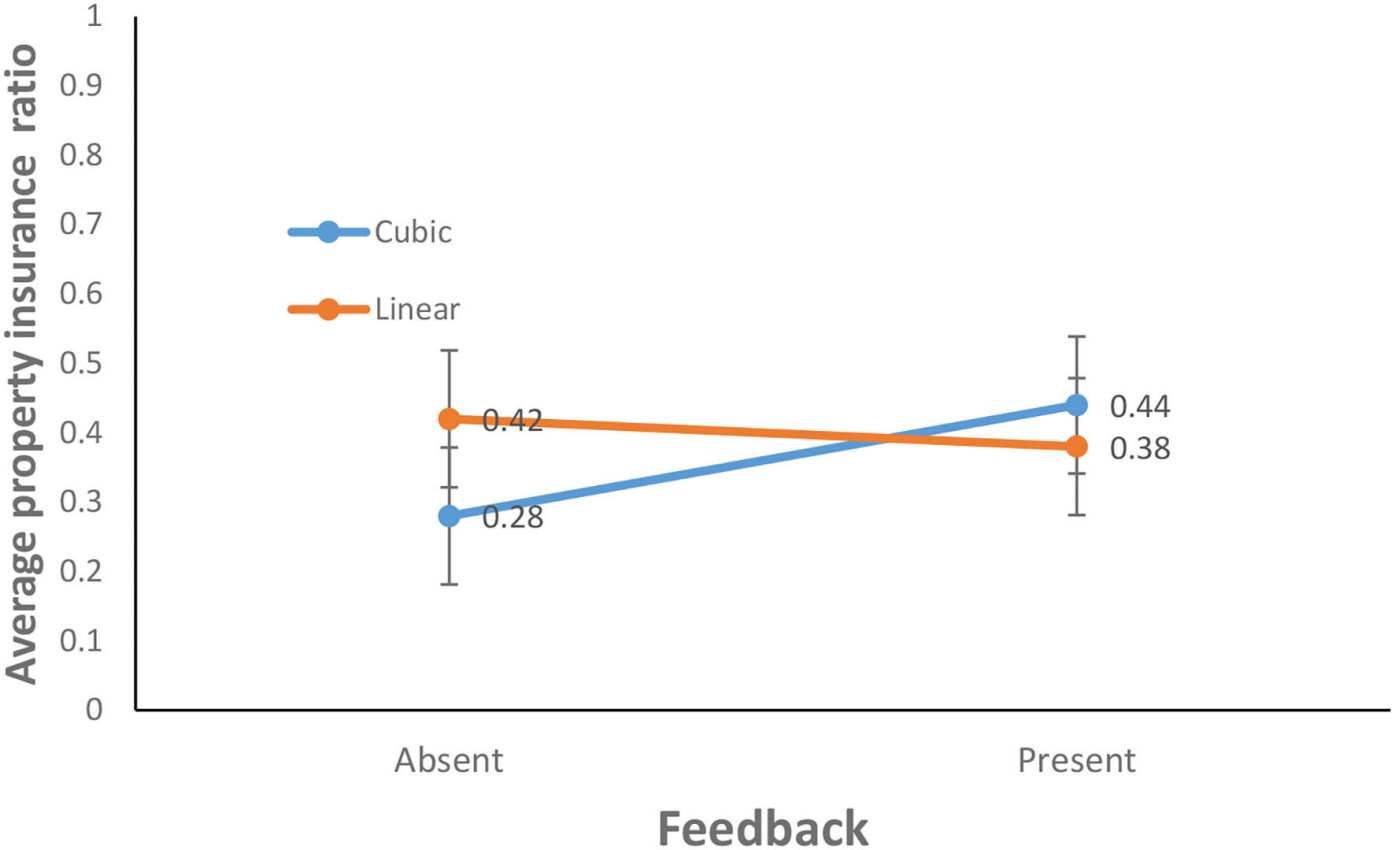
Average property insurance ratio in cubic and linear probability of climate change and feedback-present or absent conditions. The error bars show 95% CI around the point estimate.

The average property insurance ratio for the feedback-present condition (0.41) was about the same as that for the feedback-absent condition (0.35) [*F*_(1, 156)_ =1.43, *p* = 0.23, η^2^ = 0.01]. This finding does not support H1. Similarly, the average property insurance ratio for the cubic probability (0.36) was about the same as that for the linear probability (0.40) [*F*_(1, 156)_ = 0.45, *p* = 0.50, η^2^ = 0.00]. Furthermore, as shown in Figure 6, the interaction between the availability of feedback and probability of climate change was significant for average property insurance ratio [*F*_(1, 156)_ = 4.21, *p* = 0.05, η^2^ = 0.03]. This finding supports H2.

The average health insurance ratio for the feedback-present condition (0.33) was about the same as that for the feedback-absent condition (0.35) [*F*_(1, 156)_ = 0.18, *p* = 0.67, η^2^ = 0.00]. This finding does not support H1. Similarly, the average health insurance ratio for the cubic probability (0.31) was about the same as that for the linear probability (0.37) [*F*_(1, 156)_ = 1.01, *p* = 0.32, η^2^ = 0.01]. The interaction between the availability of feedback and probability of climate change was not significant for average health insurance ratio [*F*_(1, 156)_ = 0.17, *p* = 0.68, η^2^ = 0.00]. This finding does not support H2.

**Figure 7:**
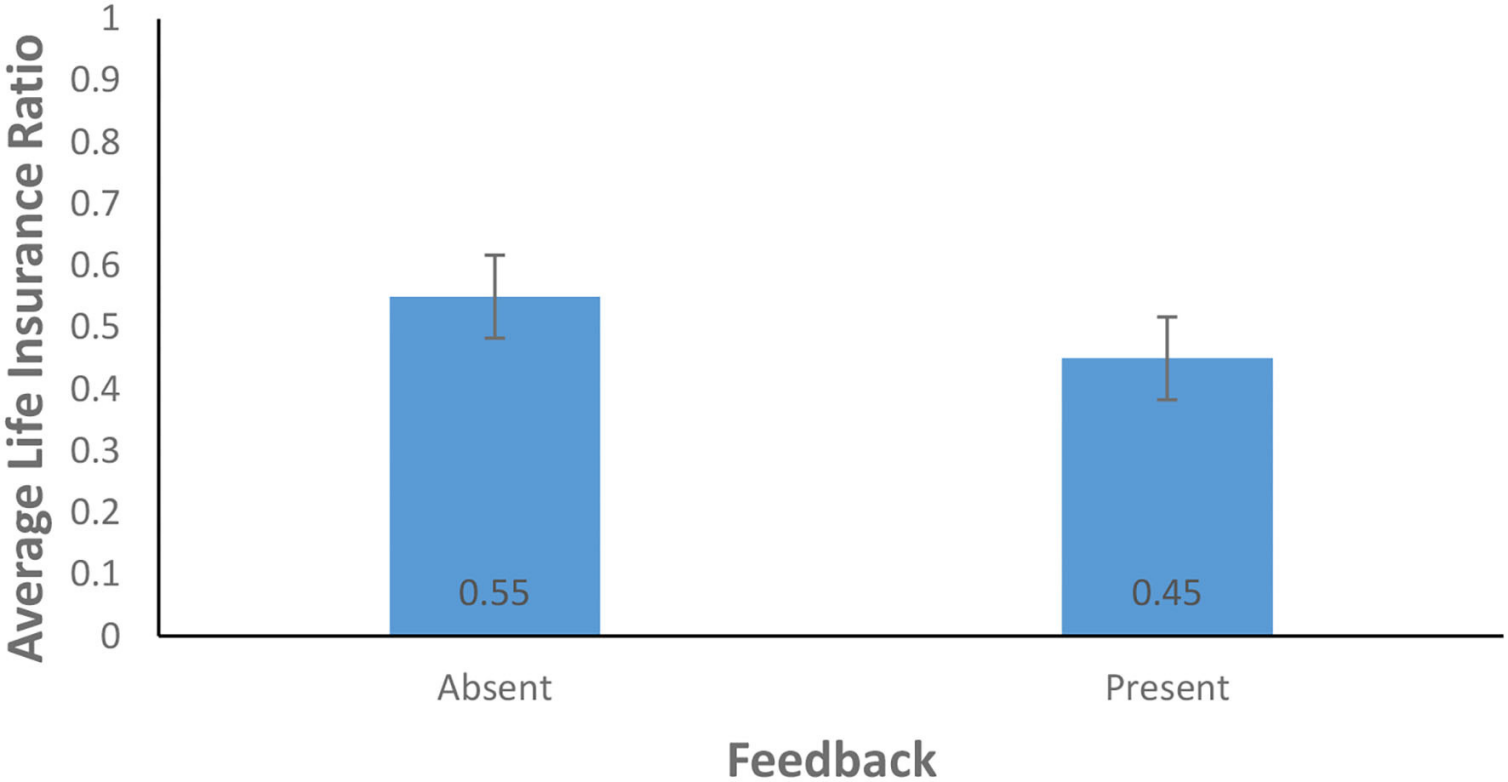
Average life insurance ratio in feedback-present and absent conditions. The error bars show 95% CI around the point estimate.

As shown in Figure 7, the average life insurance ratio for the feedback-present condition (0.45) was lower than for the feedback-absent condition (0.55) [*F*_(1, 156)_ = 3.90, *p* = 0.05, η^2^ = 0.02]. The present finding does not support H1 as the availability of feedback resulted in lower investment. In contrast, the probability of climate change did not yield a significant effect: The average life insurance ratio for the cubic probability (0.48) was about the same as that for the linear probability (0.52) [*F*_(1, 156)_ = 0.42, *p* = 0.52, η^2^ = 0.00]. The interaction between the availability of feedback and the probability of climate change was not significant for the average life insurance ratio [*F*_(1, 156)_ = 3.19, *p* = 0.08, η^2^ = 0.02]. This finding does not support H2.

### Investment Ratios Over Years

**Figure 8:**
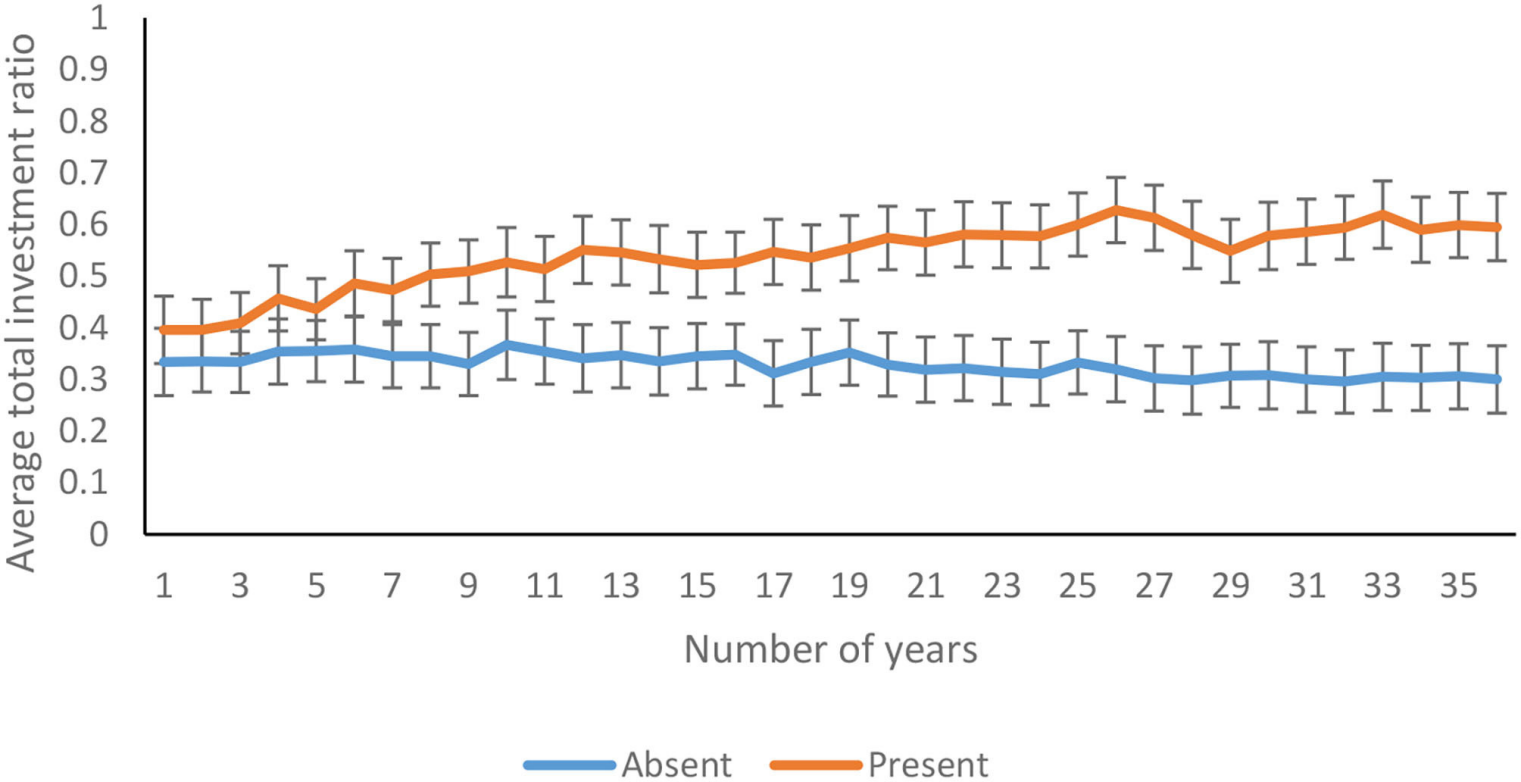
Average total investment ratio over years in feedback-present and absent conditions. The error bars show 95% CI around the point estimate.

The average total investment ratio increased significantly over 36 years [*F*_(13.76, 2146.03)_ = 3.06*, p* <0.01, η^2^ = 0.19]. As shown in Figure 8, the total investment ratio increased over 36 years in feedback-present condition; however, there was a decline in feedback-absent condition [*F*_(13.76, 2146.03)_ = 6.64*, p* <0.01, η^2^ = 0.04]. In contrast, there was an absence of interaction between the probability of climate change and trials for the total investment ratio [*F*_(13.76, 2146.03)_ = 0.46, *p* = 0.95, η^2^ = 0.00]. Furthermore, the three-way interaction between availability of feedback, probability of climate change, and trials was not significant for the total investment ratio [*F*_(13.76, 2146.03)_ = 0.53, *p* = 0.91, η^2^ = 0.00].

**Figure 9:**
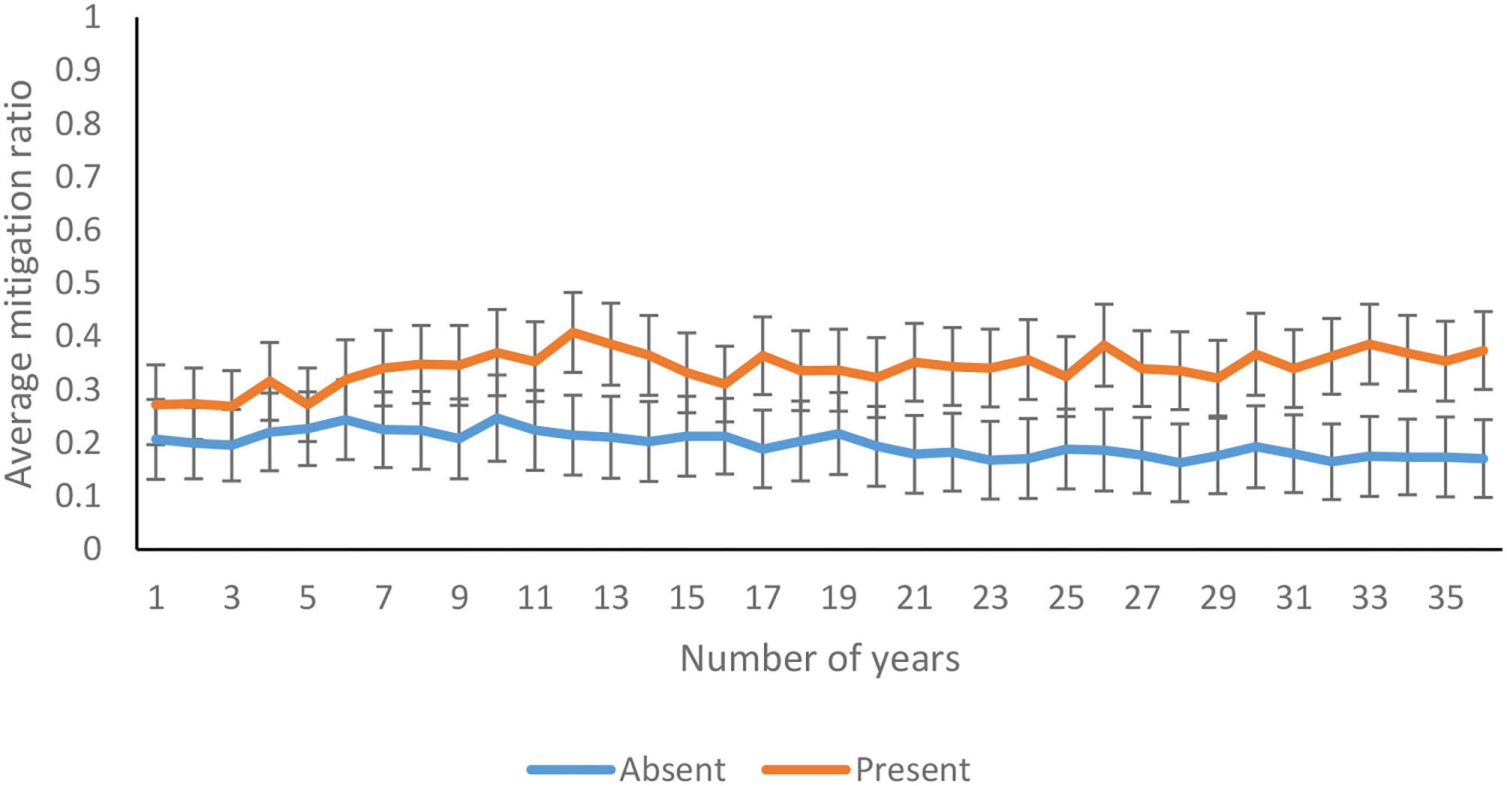
Average mitigation ratio over years in feedback-present and absent conditions. The error bars show 95% CI around the point estimate.

The average mitigation ratio was not affected significantly over 36 years [*F*_(14.04, 2189.89)_ = 1.238*, p* = 0.24, η^2^ = 0.01]. As shown in Figure 9, the mitigation ratio increased over 36 years in feedback-present conditions; however, there was a decline in feedback-absent conditions [*F*_(14.04, 2189.89)_ = 1.816*, p* = 0.03, η^2^ = 0.01]. In contrast, there was an absence of interaction between probability of climate change and trials for the mitigation ratio [*F*_(14.04, 2189.89)_ = 0.56*, p* = 0.90 η^2^ = 0.00]. Furthermore, the three-way interaction between availability of feedback, probability of climate change, and trials was not significant for the mitigation ratio [*F*_(14.04, 2189.89)_ = 0.60*, p* = 0.87, η^2^ = 0.00].

**Figure 10:**
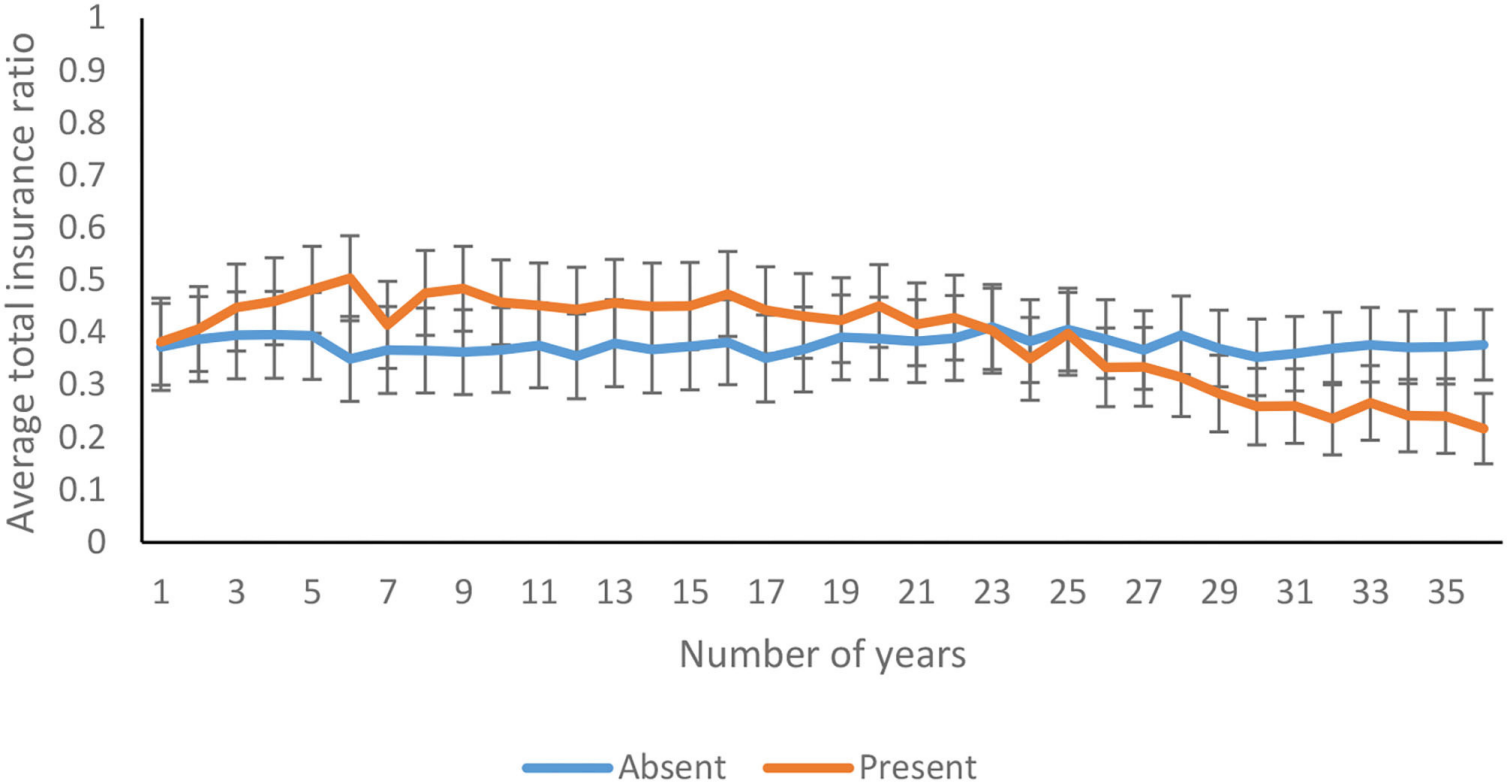
Average total insurance ratio over years in feedback-present and absent conditions. The error bars show 95% CI around the point estimate.

The average total insurance ratio decreased significantly over 36 years [*F*_(11.50, 1793.22)_ = 6.77*, p* <0.01, η^2^ = 0.04]. As shown in Figure 10, the total insurance ratio decreased over 36 years in feedback-present conditions; however, there was only a marginal variation in feedback-absent conditions [*F*_(11.50, 1793.22)_ = 6.35*, p* <0.01, η^2^ = 0.04]. In contrast, there was an absence of interaction between probability of climate change and trials for the total insurance ratio [*F*_(11.50, 1793.22)_ = 0.86*, p* = 0.58, η^2^ = 0.01]. Furthermore, the three-way interaction between availability of feedback, probability of climate change, and trials was not significant for the total insurance ratio [*F*_(11.50, 1793.22)_ = 1.00*, p* = 0.44, η^2^ = 0.01].

**Figure 11:**
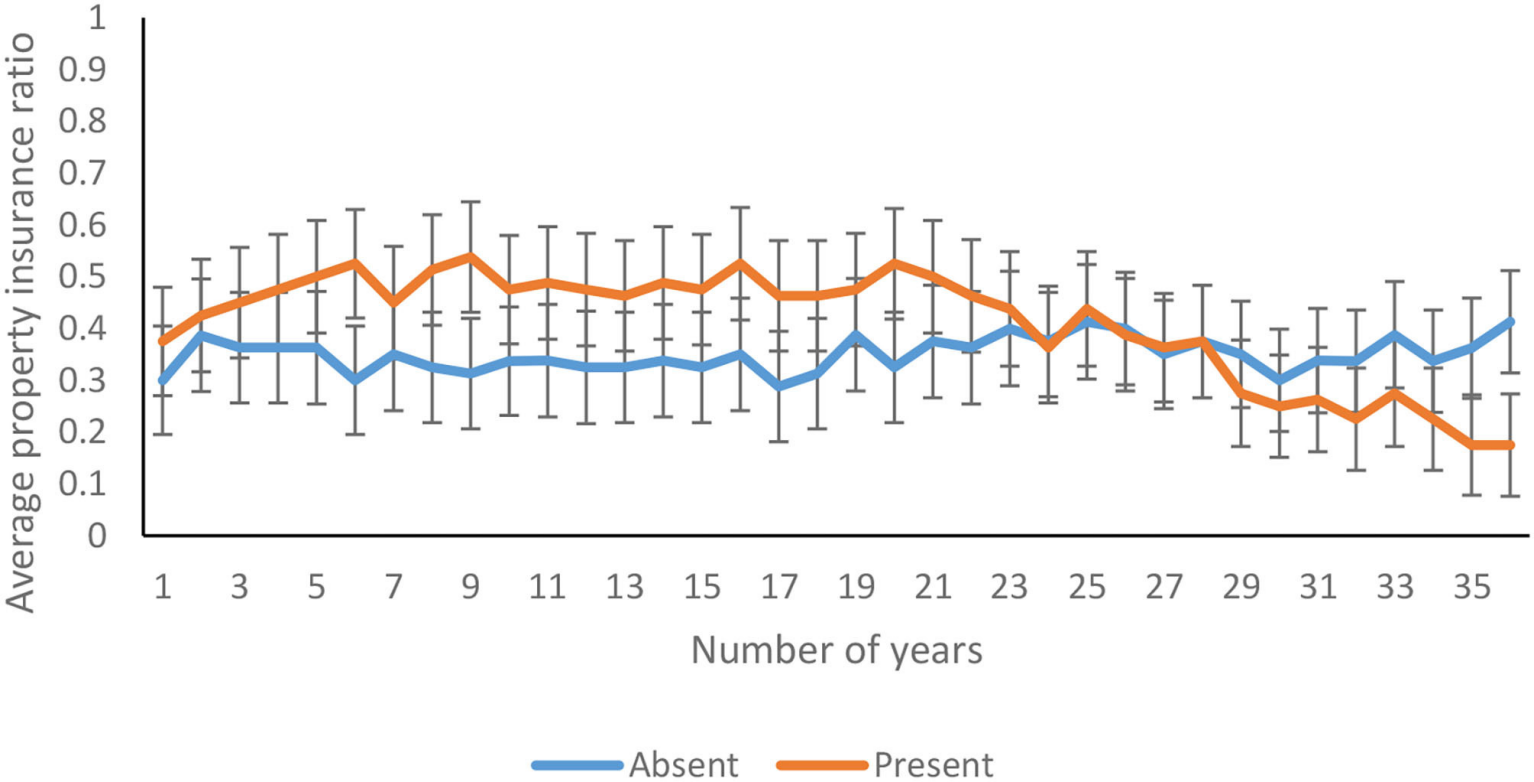
Average property insurance ratio over years in feedback-present and absent conditions. The error bars show 95% CI around the point estimate.

The average property insurance ratio decreased significantly over 36 years [*F*_(17.60, 2745.61)_ = 3.33*, p* <0.00, η^2^ = 0.02]. As shown in Figure 11, the property insurance ratio decreased over 36 years in feedback-present conditions; however, there was only a marginal variation in feedback-absent conditions [*F*_(17.60, 2745.61)_ = 4.26*, p* <0.00, η^2^ = 0.03]. In contrast, there was an absence of interaction between probability of climate change and trials for the property insurance ratio [*F*_(17.60, 2745.61)_ = 0.92*, p* = 0.56, η^2^ = 0.01]. Furthermore, the three-way interaction between availability of feedback, probability of climate change, and trials was not significant for the property insurance ratio [*F*_(17.60, 2745.61)_ = 0.79*, p* = 0.71, η^2^ = 0.01].

**Figure 12:**
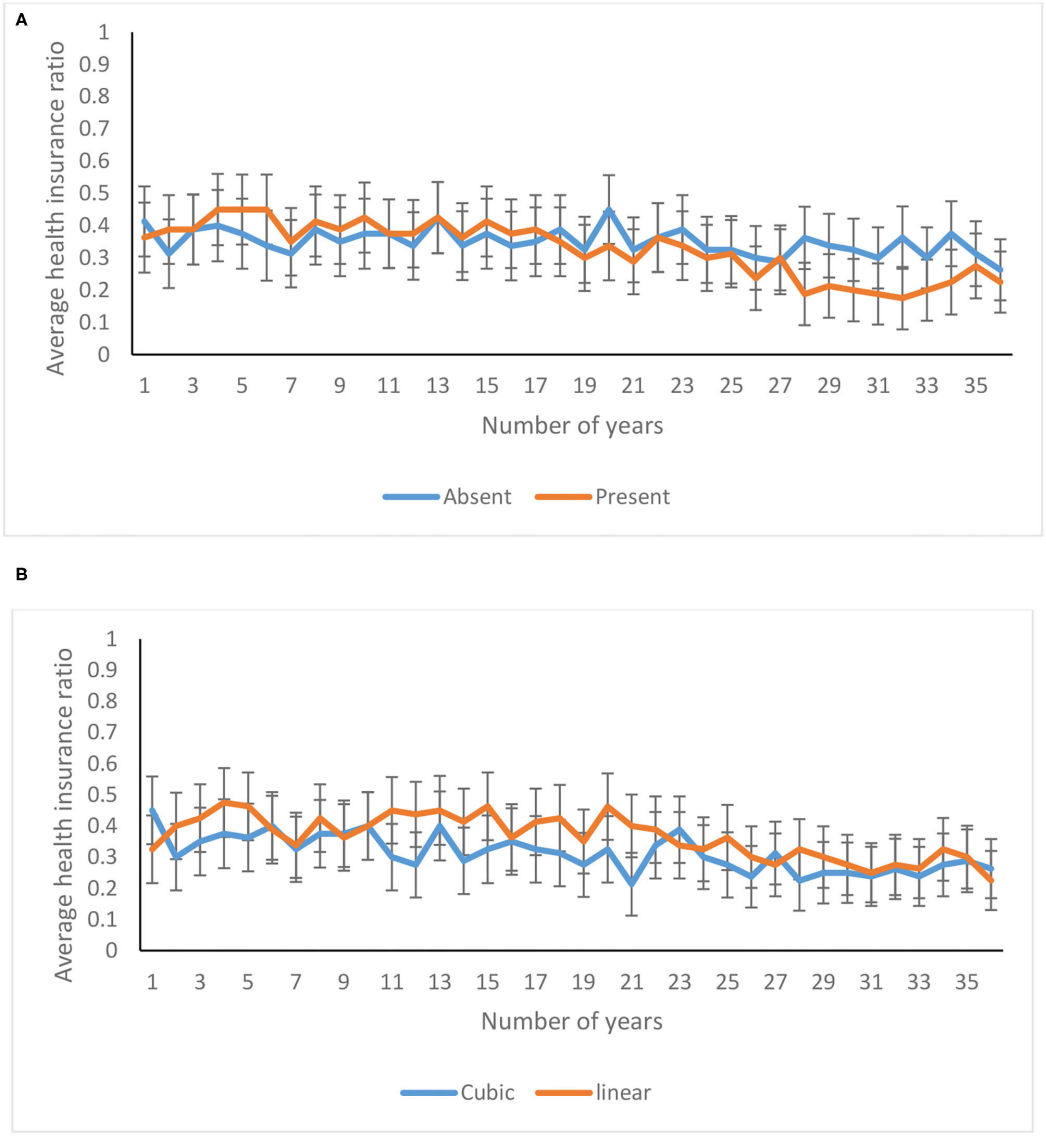
Average health insurance ratio over years in **(A)** feedback-present and absent conditions, and in **(B)** cubic and linear probability conditions. The error bars show 95% CI around the point estimate.

The average health insurance ratio decreased significantly over 36 years [*F*_(22.58, 3522.62)_ = 4.24*, p* <0.00, η^2^ = 0.03]. As shown in Figure 12A, the health insurance ratio decreased over 36 years in feedback-present conditions; however, there was only a marginal decrease in feedback-absent conditions [*F*_(22.58, 3522.62)_ = 1.92*, p* = 0.01, η^2^ = 0.01]. Similarly, as shown in Figure 12B, the health insurance ratio was higher for the linear probability, in comparison with the cubic probability, and the investment pattern in both the cubic and linear conditions was quite similar [*F*_(22.58, 3522.62)_ = 1.56*, p* = 0.05, η^2^ = 0.01]. Furthermore, the three-way interaction between availability of feedback, probability of climate change, and trials was not significant for the health insurance ratio [*F*_(22.58, 3522.62)_ = 0.94*, p* = 0.54, η^2^ = 0.01].

The average life insurance ratio was not affected significantly over 36 years [*F*_(22.24, 3469.32)_ = 1.29*, p* = 0.17, η^2^ = 0.01]. There was an absence of interaction between feedback function and trials for the life insurance ratio [*F*_(22.24, 3469.32)_ = 1.39*, p* = 0.11, η^2^ = 0.01]. Similarly, there was an absence of interaction between probability function and trials for the health insurance ratio [*F*_(22.24, 3469.32)_ = 1.00*, p* = 0.47, η^2^ = 0.01]. Furthermore, the three-way interaction between availability of feedback, probability function, and trials was not significant for the life insurance ratio [*F*_(22.24, 3469.32)_ = 1.48*, p* = 0.07, η^2^ = 0.01].

## Discussions and Conclusions

The present study aimed to understand the effect of feedback and the probability of climate change in influencing people's mitigation and adaptation decisions toward climate change. The obtained results showed that the feedback significantly affected three out of six dependent variables: total investment ratio, mitigation ratio, and life insurance ratio. The probability of climate change did not significantly affect any of the six dependent variables. However, the interaction between feedback and probability of climate change significantly affected three out of six dependent variables: total investment ratio, mitigation ratio, and property insurance ratio.

It was hypothesized that the presence of feedback would lead to greater investment in mitigation and adaptation against climate change. Among the three ratios significantly affected by the feedback function, two followed H1, and one was contrary to H1. In the presence of the feedback, participants invested significantly more in the total investment ratio and mitigation ratio compared to when it was absent. These results provide support to previous laboratory-based research on the effectiveness of induced feedback (Dutt and Gonzalez, 2012b; Chaturvedi et al., 2017; Kumar and Dutt, 2018). Based on the participants' monetary investments against climate change ICCS tool simulated the cataclysmic outcomes of climate change. Participants were able to experience these cataclysmic outcomes in the form of feedback generated by ICCS. This trial-by-trial feedback may have facilitated learning by a repeated trial-and-error procedure (Simon, 1959), and it may have resulted in an increased investment against climate change.

In contradiction to H1, feedback did not affect the following three adaption ratios: total insurance ratio, property insurance ratio, and health insurance ratio. Furthermore, the life insurance ratio did not support H1 as the availability of feedback resulted in lower life insurance investments. A likely reason for the contradictory effect or the ineffectiveness of feedback for adaption schemes could be explained based on the work done by Slovic et al. (2005). They reported that the loss-aversive individual tends to show a more significant contribution against risks over time. In the present case, something similar was observed to Slovic et al. (2005); the participants in the presence of feedback showed an increase in their investment against disasters toward the end of the game after experiencing the adverse effects and losses incurred because of those disasters.

Furthermore, they prioritized mitigating climate change instead of adapting to it. This favorability toward mitigation could have been to remove the possibility of incurring a monetary loss completely. Investment in mitigation leads to a lower probability of climate change occurrence, and in the absence of climate change, participants do not incur any losses. In contrast, investment in adaptive insurance schemes only attenuated the monetary loss incurred and did not lower the probability of climate change. Thus, an investment in adaptive insurance schemes still left the participants prone to the losses simulated due to climate change. Prioritizing climate change mitigation over adaptation in such a situation may be a smart tactic. That is because, by investing in mitigation, one could reduce the likelihood of experiencing any monetary loss. However, adaptation investments only lower the monetary loss that people might incur. Also, of the three damages in the ICCS model, fatality damage had the least probability of occurring if climate change was simulated. Participants in the feedback conditions may have realized this as the simulation progressed. Therefore, we speculate that the least probability of experiencing loss due to fatality, coupled with participant's behavior of prioritizing mitigation over adaptation, can be a likely reason for significantly lower investment in the life insurance ratio in the presence of feedback.

Another likely reason for the significantly greater investment in mitigation over adaptation could be the limited monetary assets. In the ICCS tool, participants incurred monetary losses due to climate change. Therefore, toward the end of the game, a large majority of participants were not left with enough monetary assets to invest in insurance schemes (insurance schemes required the corresponding minimum amount to enroll in them). In such a situation, mitigation was the only option available for participants since it allowed flexibilities in the amount to be invested. Overall, not possessing the necessary capital for purchasing insurance could also be a reason for the smaller adaptation investments than mitigation investments.

The probability models of climate change occurrence did not affect the monetary investments against climate change. This result could be due to the limited number of trials in the ICCS tool. Though, the cubic model may have simulated climate change on more occasions than the linear model. Still, such occasions may not be significantly higher as the present version of the ICCS tool had only 36 trials. Furthermore, as noted, there was a significantly greater investment in mitigation toward the end of the simulation in the presence of feedback. This increase in mitigation amount reduced the probability of climate change occurrence. The reduced probability of climate change due to the increase in mitigation amount, coupled with the limited number of trials, may have been the reason why the probability of climate change on its own did not significantly affect the dependent monetary variables. It is interesting to note that prior research found the probability of climate change to be a significant factor (Milinski et al., 2008). For example, in the experiment conducted by Milinski et al. (2008), the probability of simulating climate change was a static factor and was not affected by participants' investment against climate change. Climate change in CRSD was simulated with a fixed probability if a group of participants failed to reach a predetermined target. In contrast, in the current study, the probability of climate change occurrence was an endogenous factor within the ICCS model, and it was continuously altered based on the participants' investments in mitigating climate change.

To the best of our knowledge, the present experiment is the first of its kind. Through the ICCS tool, participants experienced the probability of climate change as an endogenous factor and also obtained feedback about the effects of their actions on the probabilistic occurrence of climate change. The endogenous climatic probability model could lead to significantly different results than the prior models based on the static probability of climate change (e.g., Milinski et al., 2008).

It was also hypothesized that there would be an interaction between the probability of climate consequences and the feedback. In accordance with H2, there was a significant interaction between the probability of climate change and the feedback function for three dependent variables: the total investment ratio, mitigation ratio, and the property insurance ratio. There were significant differences in investment in the total investment ratio between linear and cubic probability models in the absence of feedback; however, the differences in investments in total investment ratio between linear and cubic probability models diminished in the presence of feedback. Similarly, there were significant differences in investments in mitigation ratio between linear and cubic probability models in the absence of feedback; however, the differences in investments in mitigation ratio between linear and cubic probability models diminished in the presence of feedback. Lastly, there were significant differences in investments in property insurance ratio between linear and cubic probability models in the absence of feedback; however, the differences in investments in property insurance ratio between linear and cubic probability models diminished in the presence of feedback.

The diminished differences of cubic and linear probability functions in the presence of feedback can be explained based on prior research (Hertwig and Erev, 2009). A high-probability event gets over-weighted when the decisions are made from experience, and a low-probability event is over-weighted when decisions are made from description (Hertwig and Erev, 2009; Hertwig, 2012). In the current experiment, the linear probability of climate change accounted for the low probability of climate change. Similarly, the cubic model accounted for the high probability of climate change. As per Hertwig (2012), in the absence of feedback, when the decisions were made from the description, the linear probability of climate change likely caused a significantly greater investment than the cubic probability of climate change. However, when the high cubic probability of climate change was experienced through ICCS in the form of feedback, it resulted in investment toward climate change that was similar to those in the linear probability of climate change (Hertwig and Erev, 2009; Hertwig, 2012). That is because, perhaps, the experience of climate consequences is noisy, and this noise makes it similar across the linear and cubic probability models. As a result, the differences in monetary investments between linear and cubic probability models diminished in the presence of feedback.

However, in contradiction to H2, there was no significant interaction between the probability of climate change and feedback for total insurance ratio, health insurance ratio, and life insurance ratio. This lack of interaction can be attributed to the investments made or not made in adaption schemes. As discussed above, the investment in adaption was not significantly affected by the presence of feedback. Also, the two probability models of climate change did not significantly affect participants' investments toward different adaptation schemes. This fact was because participants prioritized mitigation over adaptation. Thus, due to the lack of investment in adaptation, the two probability models were neither able to amplify nor able to attenuate the investments made in a significant manner, whether feedback was present or absent. The results obtained for the investment over the years (trials) support these findings. The investment made in mitigation ratio in the presence of feedback increased significantly throughout trials. At the same time, there was a significant decrease in investment in the presence of feedback toward all adaption schemes, except the life insurance ratio, which was not significantly affected. Therefore, we speculate that the eventual decrease in investment toward adaption schemes, most likely at the cost of investing in mitigation, resulted in an insignificant effect of feedback on adaption strategies and eventually no significant interaction between the probability of climate consequences and feedback.

One limitation of the present paper is that our immediate goal was to study the general effects of experience and the probability of climate change on the monetary actions of people. For this purpose, we used approximate monetary values for losses motivated by similar environmental simulation tools. Therefore, the actual values for loss incurred in the real world could be different from those assumed in the ICCS. Furthermore, although the present model accounts for the different strategies (adaption and mitigation) to invest against climate change individually, other models may exist where an individual may work individually or in a group to combat climate change. Also, the present computational model only accounted for climate change's anthropogenic factors. In the real world, both human activities and naturally occurring events like volcanic eruptions and natural wildfires may contribute to GHG concentrations in the environment. Also, there may still be a possibility of disasters no matter how much was invested against climate change. The present ICCS model does not account for such stochastic and naturally occurring elements of climate change disasters. Lastly, government bodies at national, regional, and local levels may also help mitigate and adapt to the changing climatic conditions. Attending to these different model assumptions and tailoring the ICCS model for the different strata of the population could further help improve the implications of the ICCS tool.

Prior research has shown that people have many misconceptions about the climatic system of the Earth and that these misconceptions contribute toward the “wait-and-see” behavior for climate change (Sterman and Sweeney, 2007; Sterman, 2008). However, the present study's results reveal that simulation tools may provide a reasonable approximation to the experience of real-world climate change consequences, and these tools may act as an aid in improving investment patterns against climate change. In light of our present findings, we believe that the simulation tools, such as the ICCS tool, could be used as a decision aid for policymakers and the general public alike. Tools like ICCS could increase the public's understanding of climate change and its associated adverse effects and educate them about the possible preventive measures and the associated investment options. The present study is an initial step toward creating an interactive dynamic system to study investment patterns against the adverse effects of climate change.

As part of our future research, we would like to test the effects of various other system variables such as the strength of the feedback, the severity of loss incurred, and the availability of varied investment options on participants' monetary investments against climate change. Additionally, another line of research could be to study the effect of a friend's investments on an individual's investments. The efforts of an individual alone cannot affect the rate of climate change. Thus, decisions against climate change are generally taken as a group or while working with other individuals. These other individuals could be those who make up an individual's social circle and are likely to share similar consequences as the individual in case of a climatic calamity. Thus, decisions made with other individuals in a group can affect an individual's decisions. Prior research suggests that the mere information that the majority of the group members are making pro-environmental decisions can motivate individuals to do the same (Kumar and Dutt, 2019). In contrast, it may also lead individuals to become free-riders since group decisions may allow people to hide among other individuals in the group.

Furthermore, advanced feedback mechanisms like virtual reality (VR) and augmented reality (AR) can be used to generate and deliver experiences similar to real-world experiences (Kirner and Kirner, 2008). Feedback provided through VR and AR may further help reduce people's wait-and-see behavior by providing more indulging and realistic experiences of climate change. Finally, we plan to cover a larger sample of the population, comprising different strata of the population, to evaluate and further enhance the generalizability of the results. Overall, tools like the ICCS have the potential to be used for climate change education and policymaking

## Data Availability Statement

The raw data supporting the conclusions of this article will be made available by the authors, without undue reservation.

## Ethics Statement

The studies involving human participants were reviewed and approved by Ethics Committee, Indian Institute of Technology Mandi, India. The patients/participants provided their written informed consent to participate in this study.

## Author Contributions

GC contributed toward designing the experiment and carried out data collection and analyses for this work. VD was the principal investigator who developed the idea of the study and served as a constant guiding light for this work. Both authors contributed to the article and approved the submitted version.

## Conflict of Interest

The authors declare that the research was conducted in the absence of any commercial or financial relationships that could be construed as a potential conflict of interest.
