# Supplementary material for: Experience in a Climate Simulator: Influence of Probability Function and Feedback on Decisions Against Climate Change
Source: Front Psychol. 2021 Jul 14;12:674892. doi: 10.3389/fpsyg.2021.674892 (PMC8316834; doi:10.3389/fpsyg.2021.674892)
Supplement: Supplementary file 1 [file Data_Sheet_1.PDF]

Appendix

Instruction text for feedback-absent condition and linear probability of climate consequences

Imagine you are a resident of India and you with your family live on a property owned by you within the country. India as a country is prone to multiple adverse effects of climate change such as cyclones, floods, and droughts. These adverse effects of climate change can cause fatalities and injuries to you and your family. In addition, they may also damage your property and cause a loss to your property wealth.

In this task, you will be repeatedly making annual investment decisions against climate change over a period of several years. For the purpose of these investments, a fictitious currency called "EC" has been proposed. Every year, you earn EC 8760. This money is your annual income and you may use a part or whole of it for making climate change insurance investments or investments for interventions that mitigate climate change.

For example, you may buy different insurance plans against climate change from your annual income. There are three types of insurance plans namely, life, health, and property insurance. Life insurance compensates you if there is a death due to adverse effects of climate change. Health insurance compensates you if there is an injury due to adverse effects of climate change. Property insurance compensates you if there is property damage due to adverse effects of climate change.

In contrast, you may decide to invest your annual income into mitigation measures against climate change. The money you invest in mitigation will be used for restoring forests, removing carbon-dioxide from the atmosphere, reducing carbon emissions, and developing a number of eco-friendly technologies.

In any year, the chance of climate change is computed by the following equation:

$$\mathrm{p} = 1 - 0.85 * \left(\frac{\sum_{\mathrm{t}=1}^{\mathrm{n}} \mathrm{investment}_{\mathrm{t}}}{\sum_{\mathrm{t}=1}^{\mathrm{n}} \mathrm{income}_{\mathrm{t}}}\right)$$

Where, p is the chance of climate change; $\sum_{t=1}^{n} \mathrm{investment}_t$ are the sum of investments made in mitigating climate change between the first year and the current (n^{th}) year; and, $\sum_{t=1}^{n} \mathrm{income}_t$ is the sum of income available to you for making investments between the first year and the current (n^{th}) year. For example, if your investment was EC 0 and EC 4380 in mitigation in the first two years and your income in each year was EC 8760, then the chance of climate change would be 0.79 = 1 – 0.85 * (0+4380) / (8760+8760)).

Your total wealth at any point in the task is the following: sum of your income that you did not put into mitigation or use for buying insurance + your property wealth - damages to you, your family, and to your property due to climate change. Your property wealth is assumed to be EC 50,00,000 at the start of the task. The income used for mitigating climate change or for buying insurance does not contribute to your total wealth.

If climate change occurs in a year, then there may be adverse effects (e.g., cyclones, floods, and droughts). These adverse effects may cause injuries, fatalities, or property damages (all these three could occur independently of the others). Injury and fatality to you or your family due to adverse effects may cause a decrease of your yearly income if health and life insurance are not bought by you for the respective year. Similarly, property damage due to adverse effects may cause a decrease of your property wealth if property insurance is not bought by you for the respective year.

If you did not buy life or health insurance and one of the adverse effects due to climate change took place, then your annual income may be reduced by 25% in case of fatality (no life insurance) and by 12.5% in case of injury (no health insurance) of its current value. Similarly, if you did not buy property insurance and cyclones or floods took place, then your property wealth may be reduced by 50% of its current value. The reduction in income and property wealth due to fatalities, injuries, and property damages are permanent and remain for the duration of the task.

Your goal is to decide how much to invest annually into mitigation and for buying insurance to maximize your total wealth (sum of your income that you did not put into mitigation or use for buying insurance + your property wealth - damages to you, your family, and to your property due to climate change). You will be compensated for completing the game at the rate initially advertised. You will only be eligible for compensation if you have completed the game in full and supplied the appropriate confirmation code. There is no partial payment if you do not complete the study. You will not be penalized if you choose to withdraw from the study without completing it, but you will not be compensated either.

BEST OF LUCK!

Online Link: http://landslidemonitoring.esy.es/gitanshu/climatechange3/instruction.php

Instruction text for feedback-present condition and linear probability of climate consequences

Imagine you are a resident of India and you with your family live on a property owned by you within the country. India as a country is prone to multiple adverse effects of climate change such as cyclones, floods, and droughts. These adverse effects of climate change can cause fatalities and injuries to you and your family. In addition, they may also damage your property and cause a loss to your property wealth.

In this task, you will be repeatedly making annual investment decisions against climate change over a period of several years. For the purpose of these investments, a fictitious currency called "EC" has been proposed. Every year, you earn EC 8760. This money is your annual income and you may use a part or whole of it for making climate change insurance investments or investments for interventions that mitigate climate change.

For example, you may buy different insurance plans against climate change from your annual income. There are three types of insurance plans namely, life, health, and property insurance. Life insurance compensates you if there is a death due to adverse effects of climate change. Health insurance compensates you if there is an injury due to adverse effects of climate change. Property insurance compensates you if there is property damage due to adverse effects of climate change.

In contrast, you may decide to invest your annual income into mitigation measures against climate change. The money you invest in mitigation will be used for restoring forests, removing carbon-dioxide from the atmosphere, reducing carbon emissions, and developing a number of eco-friendly technologies. In any year, the chance of climate change is determined by your investments for mitigating climate change between the first year and the current year.

Your total wealth at any point in the task is the following: sum of your income that you did not put into mitigation or use for buying insurance + your property wealth - damages to you, your family, and to your property due to climate change. Your property wealth is assumed to be EC 50,00,000 at the start of the task. The income used for mitigating climate change or for buying insurance does not contribute to your total wealth.

Once you have made your investment decisions (for buying insurance or for mitigating climate change), you will be provided feedback about whether climate change occurred or not. If climate change occurs in a year, then there may be adverse effects (e.g., cyclones, floods, and droughts). These adverse effects may cause injuries, fatalities, or property damages (all these three could occur independently of the others). Injury and fatality to you or your family due to adverse effects may cause a decrease of your yearly income if health and life insurance are not bought by you for the respective year. Similarly, property damage due to adverse effects may cause a decrease of your property wealth if property insurance is not bought by you for the respective year.

If you did not buy life or health insurance and one of the adverse effects due to climate change took place, then your annual income may be reduced by 25% in case of fatality (no life insurance) and by 12.5% in case of injury (no health insurance) of its current value. Similarly, if you did not buy property insurance and cyclones or floods took place, then your property wealth may be reduced by 50% of its current value. The reduction in income and property wealth due to fatalities, injuries, and property damages are permanent and remain for the duration of the task.

Your goal is to decide how much to invest annually into mitigation and for buying insurance to maximize your total wealth. You will be compensated for completing the game at the rate initially advertised. You will only be eligible for compensation if you have completed the game in full and supplied the appropriate confirmation code. There is no partial payment if you do not complete the study. You will not be penalized if you choose to withdraw from the study without completing it, but you will not be compensated either.

BEST OF LUCK!

Online Link: http://landslidemonitoring.esy.es/gitanshu/climatechange1/instruction.php
